# Mast cell repopulation of the peritoneal cavity: contribution of mast cell progenitors versus bone marrow derived committed mast cell precursors

**DOI:** 10.1186/1471-2172-11-32

**Published:** 2010-06-24

**Authors:** Maria Célia Jamur, Andréa N Moreno, Luciana FC Mello, Devandir A Souza Júnior, Maria Rita C Campos, Maria Verônica D Pastor, Ana Cristina G Grodzki, Deise C Silva, Constance Oliver

**Affiliations:** 1 Department of Cell and Molecular Biology and Pathogenic Bioagents, Faculdade de Medicina de Ribeirão Preto, University of São Paulo, Ribeirão Preto, Brazil; 2 Centro de Ciências Biológicas e da Saúde, Pontifícia Universidade Católica do Paraná, Curitiba, Brazil; 3 Centro de Ciências da Saúde, Universidade do Vale do Itajaí-UNIVALI, Itajaí, Brazil

## Abstract

**Background:**

Mast cells have recently gained new importance as immunoregulatory cells that are involved in numerous pathological processes. One result of these processes is an increase in mast cell numbers at peripheral sites. This study was undertaken to determine the mast cell response in the peritoneal cavity and bone marrow during repopulation of the peritoneal cavity in rats.

**Results:**

Two mast cell specific antibodies, mAb AA4 and mAb BGD6, were used to distinguish the committed mast cell precursor from more mature mast cells. The peritoneal cavity was depleted of mast cells using distilled water. Twelve hours after distilled water injection, very immature mast cells could be isolated from the blood and by 48 hours were present in the peritoneal cavity. At this same time the percentage of mast cells in mitosis increased fourfold. Mast cell depletion of the peritoneal cavity also reduced the total number of mast cells in the bone marrow, but increased the number of mast cell committed precursors.

**Conclusions:**

In response to mast cell depletion of the peritoneal cavity, a mast cell progenitor is released into the circulation and participates in repopulation of the peritoneal cavity, while the committed mast cell precursor is retained in the bone marrow.

## Background

Mast cells are known to play a pivotal role in inflammatory and allergic reactions. Recently, they have gained new importance as immunoregulatory cells with the recognition that they are a major source of cytokines and chemokines and play roles in both innate and adaptive immunity [1-3]. Despite their growing significance in normal and pathological conditions, much still remains to be learned about mast cell recruitment and maturation. Like blood cells, mast cells are derived from pluripotent hematopoietic stem cells, but unlike blood cells they leave the bone marrow as progenitors and migrate to peripheral sites where they complete their maturation [4-6]. Mast cell numbers increase at peripheral sites in response to inflammatory or allergic processes as well as in response to pathogens [7-9]. This increase in mast cell number is thought to be the result of proliferation of resident mast cell progenitors (MCp) as well as the recruitment of MCp from the bone marrow [10-14]. Recent studies from our laboratory have identified a committed mast cell precursor (MCcp) present in mouse bone marrow that is distinct from the tissue MCp [15]. In the previous study, a subtractive immunomagnetic isolation procedure with two mast cell specific antibodies, mAb AA4 and mAb BGD6, was used to purify the MCcp from mouse bone marrow. mAb AA4 recognizes two derivatives of the ganglioside GD1b that are unique to rodent mast cells [15-19], while mAb BGD6 binds to a 110 kDa protein on the surface of rodent mast cells [15,20]. Both mAb AA4 [18] and mAb BGD6 bind to granulated mast cells in all stages of maturation, but mAb BGD6 also binds to an undifferentiated cell in the bone marrow that is not recognized by mAb AA4. This undifferentiated cell was characterized as a MCcp [15]. The present study was undertaken to determine the mast cell response in the peritoneal cavity and the bone marrow during repopulation of the peritoneal cavity in rats. It was of interest to determine whether the MCp or the MCcp was involved in repopulation of the peritoneal cavity. The results of the present study demonstrate that in response to mast cell depletion of the peritoneal cavity, a MCp is released into the circulation and migrates to the peritoneal cavity, while the MCcp is retained in the bone marrow.

## Results

### Mast cell depletion of the peritoneal cavity reduces the mast cell number in bone marrow

**Figure 1:**
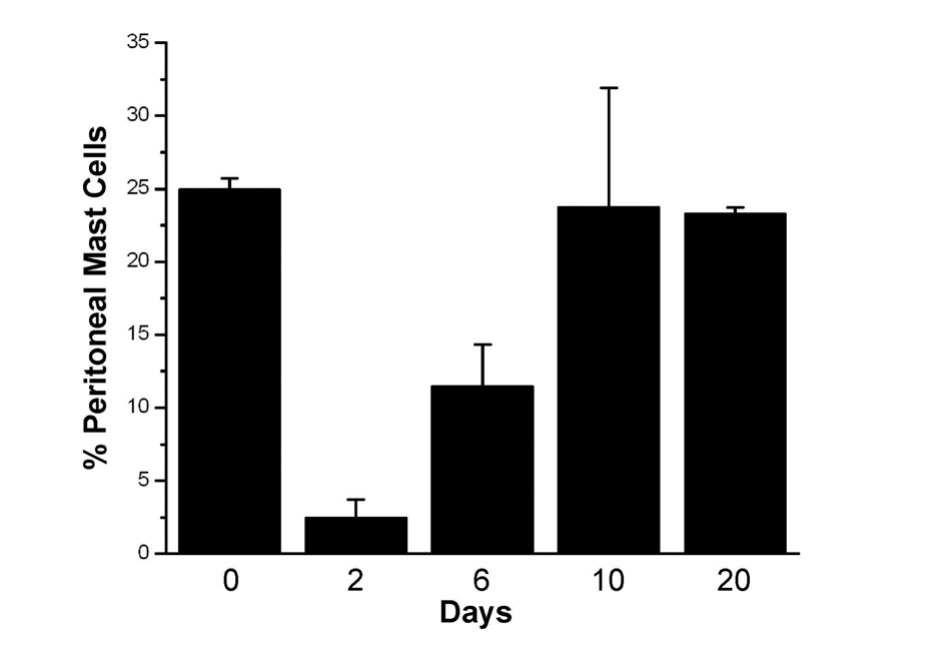
**Injection of distilled water into the peritoneal cavity results in a rapid depletion of mast cells **. Two days after injection of distilled water MCp begin to repopulate the peritoneal cavity and by 10 days the repopulation is virtually complete. Mast cells were isolated from the peritoneal lavage by magnetic beads conjugated to mAb AA4. The number of cells free or attached to the magnetic beads was determined by counting the cells in a hemocytometer. The average of 6 independent experiments ± SD is shown.

**Figure 2:**
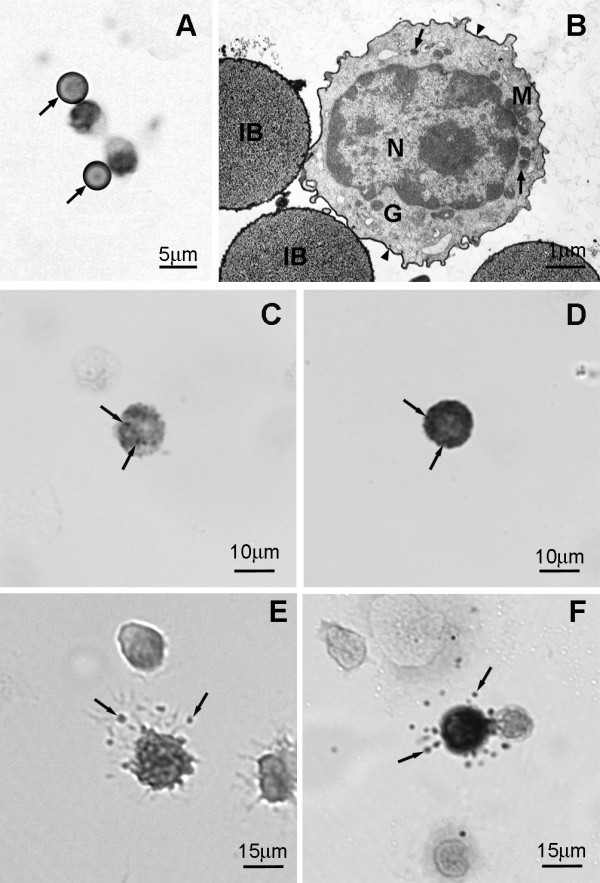
**On day 2 following i.p. injection of distilled water only MCp are present in the peritoneal lavage **. The MCp were isolated using magnetic beads conjugated to mAb AA4. (A) By light microscopy, the cells, which can be recognized by their attachment to immunomagnetic beads (arrows), have a large nucleus and no metachromatic granules. Toluidine blue. (B) By electron microscopy the MCp has a large centrally located nucleus (N), a few small secretory granules (arrows), and a compact Golgi complex (G). The cell surface also stains positively with anti-IgE (arrowheads). IB, immunomagnetic bead; M, mitochondria. Immature mast cells are present in the peritoneal lavage at 6 and 10 days after distilled water injection. (C) Representative immature peritoneal mast cell 6 days after i.p. injection of distilled water contains a few metachromatic granules (arrows). Toluidine blue. (D) Peritoneal mast cell, representative of the peritoneal mast cell population 10 days after i.p. injection of distilled water, contains many metachromatic granules (arrows). Toluidine blue. (E) Representative immature peritoneal mast cell 6 days after i.p. injection of distilled water is degranulated after exposure to antigen (arrows, released secretory granule matrix). Toluidine blue. (F) 10 days after i.p. injection of distilled water, this representative peritoneal mast cell, is degranulated after exposure to antigen. (arrows, released secretory granule matrix). Toluidine blue.

Intraperitoneal injection of distilled water is well known to lyse mast cells resulting in their disappearance [21-28]. In order to examine the kinetics of mast cell repopulation of the peritoneal cavity following distilled water lysis, mast cells were immunomagnetically separated from the peritoneal lavage using either mAb AA4 or mAb BGD6 conjugated to magnetic beads. In non depleted animals mast cells comprise 25% ± 0.73% of the total cells in the peritoneal lavage (Fig. 1). These mast cells are replete with metachromatic granules and are AA4+/BGD6+ [15,18,20]. By 2 days after distilled water injection, although repopulation of the peritoneal cavity has begun, the per cent of mast cells in the lavage is only 2.5% ± 0.77% and is composed of very immature mast cells with characteristics consistent with their identification as MCp. By light microscopy, these MCp have a large nucleus and no metachromatic granules (Fig 2A). The MCp isolated from the peritoneal fluid 48 hours after injection of distilled water could be conclusively identified as mast cells only by immunostaining in combination with transmission electron microscopy. These mast cells contain a few small cytoplasmic granules, a poorly developed Golgi complex, few mitochondria and bind IgE (Fig 2B). By immunostaining, more than 98% of these MCp also express the α subunit of FcεRI, c-kit, CD34 and CD13 and express the mast cell specific gangliosides recognized by mAb AA4 (Table 1). At six days after distilled water injection the number of mast cells in the peritoneal lavage increases to 11.5% ± 1.29% and by ten days the per cent of mast cells in the peritoneal lavage is close to control values, 23.75% ± 3.51%. However, there is more variability among the animals at 10 days, reflecting individual differences in the rate of mast cell repopulation of the peritoneal cavity. The mast cells isolated from the peritoneal lavage six (Fig. 2C) and ten days (Fig. 2D) after distilled water lysis, are more mature and can be recognized by light microscopy by the presence of metachromatic granules. When stimulated by antigen, at 6 days (Fig. 2E) and 10 days (Fig. 2F) after distilled water injection, the mast cells from the peritoneal cavity degranulate. These immature mast cells also degranulate in the presence of Compound 48/80 or when FcεRI is crosslinked by mAb BC4 (data not shown), thus demonstrating their functional capability. By 20 days, repopulation is complete and the per cent of mast cells is almost the same, 23.3% ± 0.42%, as in non depleted animals.

**Table 1:** Phenotype of MCcp, MCp and Mature Mast Cells (MC)

	Bone Marrow		Blood	Peritoneal Cavity	
	
	*MCcp*	MCp*	*MCp*	*MCp*	*MC*
*Antibody:*					
**mAb BGD6 **	**+ **	**+ **	**+ **	**+ **	**+ **

**mAb AA4 **	-	**+ **	**+ **	**+ **	**+ **

**Anti-FcεRI **	-	**+ **	**+ **	**+ **	**+ **

**Anti-IgE **	-	**+ **	**+ **	**+ **	**+ **

**Anti c-kit **	**+ **	**+ **	**+ **	**+ **	**+ **

**Anti-CD13 **	**+ **	**+ **	**+ **	**+ **	**+ **

**Anti-CD34 **	**+ **	**+ **	ND*	**+**	**±**

***Diameter***	**3-4 μm **	**3-6 μm **	**3-6 μm **	**3-6 μm **	**10-20 μm **

*[18]

+ = > 98% of the cells are positive

± = 38% of the cells are positive

- = no immunostaining was detected

Cells were immunostained with the different antibodies in 7 different experiments. In each experiment a minimum of 500 cells per antibody were counted by fluorescence microscopy.

**Figure 3:**
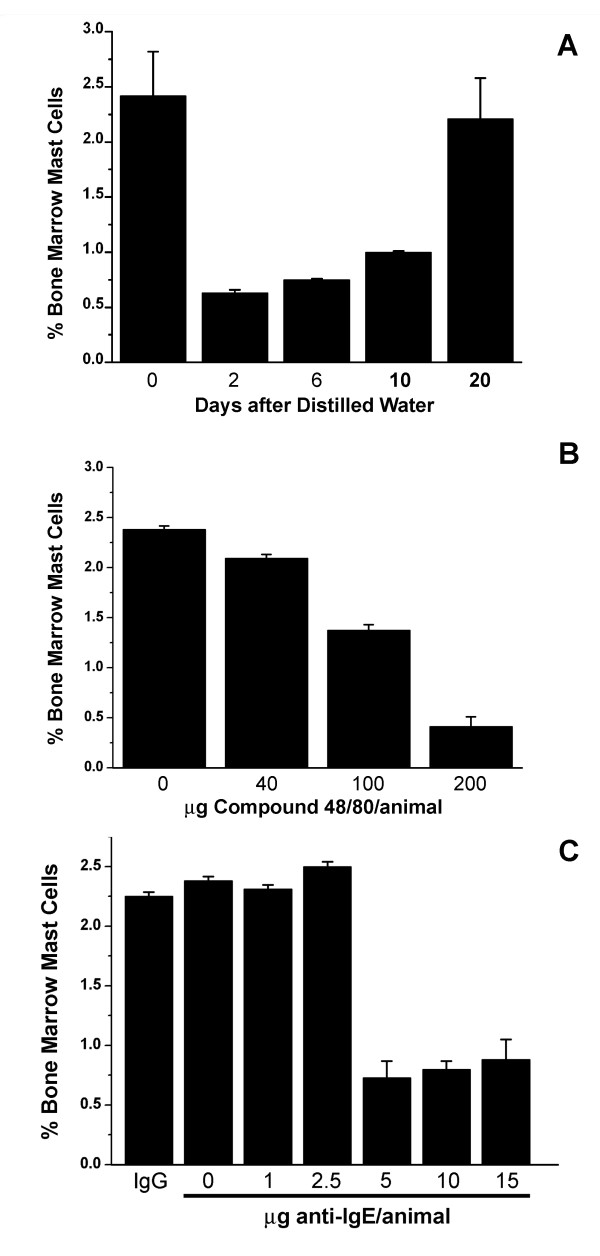
**Release of mast cell mediators in the peritoneal cavity affects bone marrow mast cell numbers **. (A) Mast cell depletion of the peritoneal cavity by distilled water results in an immediate reduction in the percentage of mast cells in the bone marrow. (B) Two days after i.p. injection of Compound 48/80 the percentage of mast cells in the bone marrow is reduced in a dose dependent manner. (C) Two days after i.p injection of anti-IgE in concentrations above 2.5 μg/animal, but not IgG the percentage of mast cells in the bone marrow is decreased. Mast cells were isolated from the bone marrow using magnetic beads conjugated to mAb BGD6. The number of cells free or attached to the magnetic beads was determined by counting the cells in a hemocytometer. The average of 3 independent experiments ± SD is shown.

If mast cells are being recruited from the bone marrow to repopulate peripheral sites, then the mast cell number should be diminished in the bone marrow after mast cell depletion of the peritoneal cavity. Indeed, repopulation of the peritoneal cavity by mast cells after distilled water injection is initially accompanied by a dramatic decrease in the number of mast cells in the bone marrow (Fig. 3A). Two days after i.p. injection of distilled water the number of AA4+/BGD6+ mast cells in the bone marrow has fallen from 2.42% ± 0.4% to 0.63% ± 0.03%. At six days the per cent of these mast cells in the bone marrow is recovering and has increased to 0.75% ± 0.01% and by 10 days has reached 1% ± 0.01%, but does not approach normal levels until day 20 (2.21 ± 0.37) when mast cell repopulation of the peritoneal cavity is complete. The number of mast cells is also reduced in a dose dependent fashion 2 days after i.p. injection of Compound 48/80 (Fig. 3B) or anti-IgE (Fig. 3C), but this reduction is not seen with IgG (Fig. 3C), indicating that the reduction in mast cell numbers in the bone marrow is a generalized response to mast cell mediator liberation in the peritoneal cavity.

**Figure 4:**
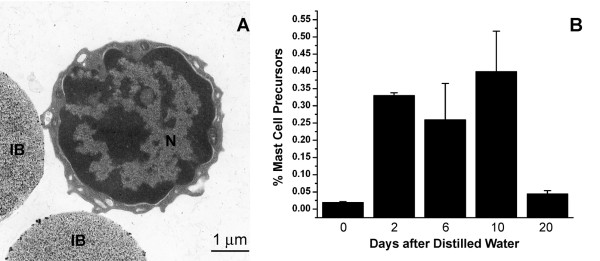
**MCcp increase in the bone marrow following mast cell depletion of the peritoneal cavity **. MCcp were isolated from bone marrow using sequential isolation with mAb AA4 conjugated magnetic beads followed by mAb BGD6 conjugated magnetic beads as detailed in the Materials and Methods. (A) By electron microscopy this representative MCcp (AA4-/BGD6+) is a small undifferentiated cell with scant cytoplasm and a large nucleus (N). (B) Lysis of peritoneal mast cells results in an increase in the percentage of AA4-/BGD6+ MCcp in the bone marrow. The average of 6 independent experiments ± SD is shown.

A MCcp, similar to that described for mouse bone marrow [15], was also identified in rat bone marrow by sequential immunomagnetic isolation with magnetic beads conjugated to mAb AA4 and mAb BGD6. By light microscopy, the AA4-/BGD6+ mast cells appear as a homogeneous population of small, 3-4 μm, undifferentiated cells with a centrally located nucleus?, little cytoplasm and contain no metachromatic granules. Electron microscopy confirmed that the isolated MCcp was a pure population of small undifferentiated cells containing scant cytoplasm, few organelles, no cytoplasmic granules and the cell surface is covered with short microvilli (Fig. 4A). The MCcp comprises 0.02% of the total cell population in rat bone marrow. By immunostaining, these cells are AA4-/BGD6+, c-kit+, CD34+, do not express FcεRI or bind IgE (Table 1). In contrast to the more mature mast cells, the per cent of AA4-/BGD6+ MCcp increases significantly from 0.02% ± 0.002% to 0.33% ± 0.008% in the first 48 hours after i. p. injection of distilled water. At six days, the number of MCcp is 0.26% ± 0.105%, and reaches 0.4% ± 0.117% by 10 days. By 20 days the number of MCcp in the bone marrow is close to normal values (0.045% ± 0.009%) (Fig. 4B). These results suggest that MCp are being recruited from the bone marrow to the peritoneal cavity and that the number of MCcp in the bone marrow increases in response to the recruitment of MCp to the peritoneal cavity.

### Repopulation of the peritoneal cavity is due to both recruitment and proliferation of mast cell progenitors

**Figure 5:**
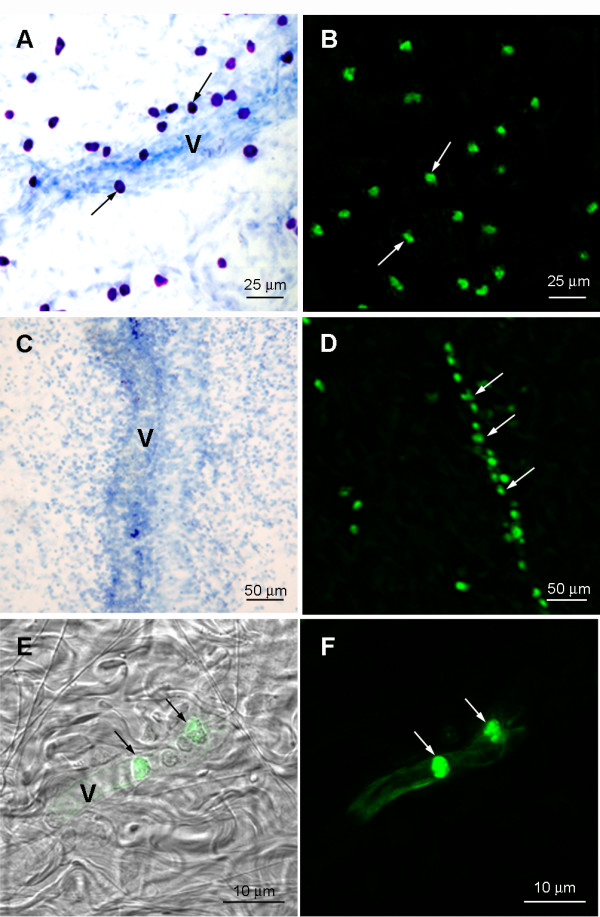
**Mast cell progenitors are present in mesenteric blood vessels 48 hours after IP injection of distilled water **. (A) In animals not injected with distilled water, mast cells (arrows) can be seen along a blood vessel (V) and in the mesenteric windows. (B) The mast cells (arrows) are positive when immunolabeled with mAb AA4-FITC (arrows). (C) No mast cells are seen in the mesentery or along a mesenteric blood vessel (V) 48 hours after distilled water injection. Toluidine blue. (D) MCp (arrows), detected by mAb AA4-FITC, are associated with and inside blood vessels (arrows). (E) Superimposition of MCp in a blood vessel immunolabeled with mAb AA4-FITC (arrows) seen in F with corresponding DIC image. Other blood cells, which do not label with mAbAA4, are also present inside the blood vessel. (F) At this magnification, MCp labelled with mAb AA4-FITC are clearly seen inside a mesenteric blood vessel.

**Figure 6:**
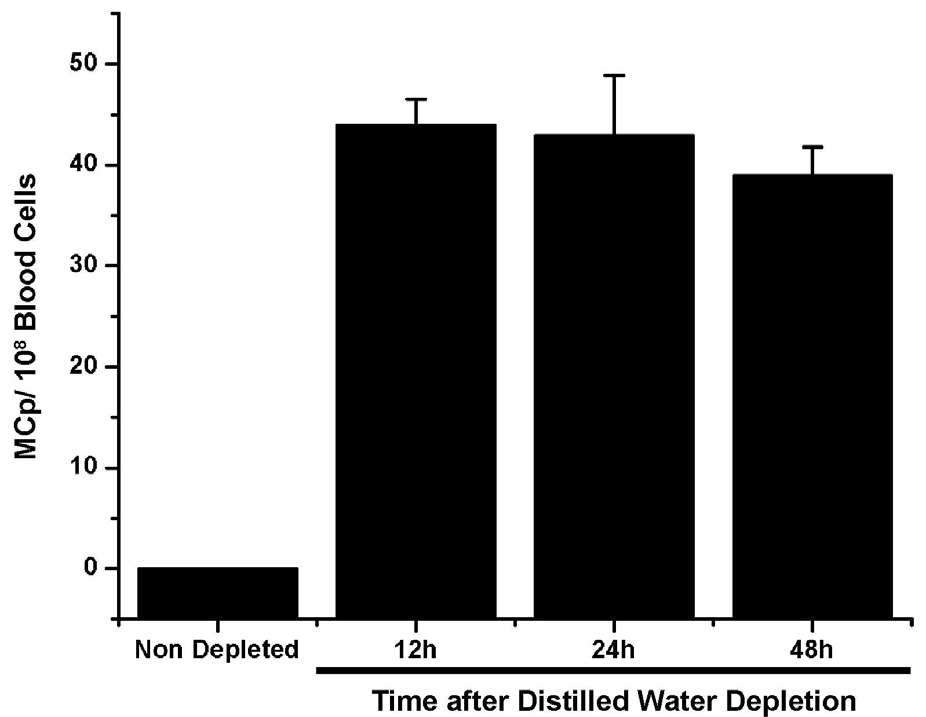
**MCp could be immunomagnetically isolated from the blood after mast cell depletion of the peritoneal cavity **. Blood was collected by cardiac puncture from nondepleted animals and animals whose peritoneal cavity had been depleted of mast cells by distilled water. By 12 hours after depletion it was possible to isolate MCps from cardiac blood. Only MCps could be isolated from cardiac blood using immunomagnetic beads conjugated to mAbBGD6. Mast cells were isolated from the blood using magnetic beads conjugated to mAb BGD6. The number of cells free or attached to the magnetic beads was determined by counting the cells in a hemocytometer. A minimum of 10 animals were used per time point. Data is expressed as the number of mast cells/10^8 ^blood cells ± S.D.

In order to further investigate the process of repopulation of the peritoneal cavity, 48 hours after distilled water injection, the mesenteries were removed and examined for the presence of mast cells. In non-depleted mesenteries mast cells could be seen along blood vessels and in the mesenteric windows. These mast cells stained with toluidine Blue (5A) and immunolabeled with either mAb AA4 (5B) or mAb BGD6. In contrast, no mast cells could be seen in the mesentery of depleted animals by toluidine blue staining (Fig. 5C). However, when the mesentery was immunostained with mAb AA4-FITC, MCp could be seen associated with mesenteric blood vessels (Fig. 5D). At higher magnification, it is clear that the MCp are inside the blood vessels (Figs. 5E and 5F). A small number of MCp, <1 mast cell/10^6 ^blood cells, could also be immunomagnetically isolated from circulating blood 12, 24 and 48 hours after mast cell depletion of the peritoneal cavity (Fig. 6). No mast cells could be isolated from the blood of non depleted animals. The MCp from the blood are morphologically and phenotypically similar to the MCp found in the bone marrow [18] as well as to the mature mast cells in the peritoneal cavity (Table 1). At no time point could MCcp (AA4-/BGD6+) be isolated from cardiac blood.

**Figure 7:**
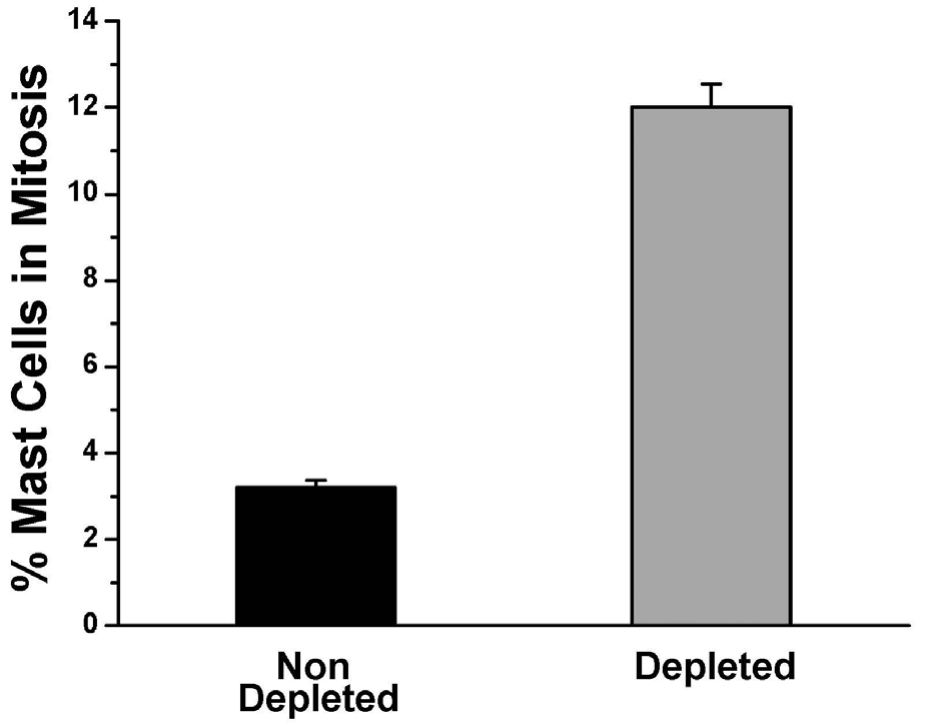
**Forty eight hours after mast cell depletion of the peritoneal cavity, there is a greater number of MCp in mitosis **. The peritoneal lavage was collected from rats depleted or non-depleted of mast cells by distilled water injection. The total cell population was double immunostained with both anti-phospho-histone H3 and mAb AA4 in order to identify mast cells in mitosis. The average of 3 independent experiments ± SD is shown.

Another factor that may contribute to the increased number of peritoneal mast cells is the proliferation of MCp recruited to the peritoneal cavity. In order to investigate this possibility, 48 hours after distilled water injection the peritoneal lavage was collected and immunostained with both anti-phospho-histone H3 and mAb AA4. In the animals that had received distilled water, there was a significant increase in the percentage of mast cells in mitosis (Fig. 7). These data indicate that it is a combination of the recruitment of MCp to the peritoneal cavity as well as the proliferation of these progenitors that results in mast cell repopulation of the peritoneal cavity after depletion by distilled water.

### Infused mast cell precursors (AA4-/BGD6+) home preferentially to the bone marrow

**Figure 8:**
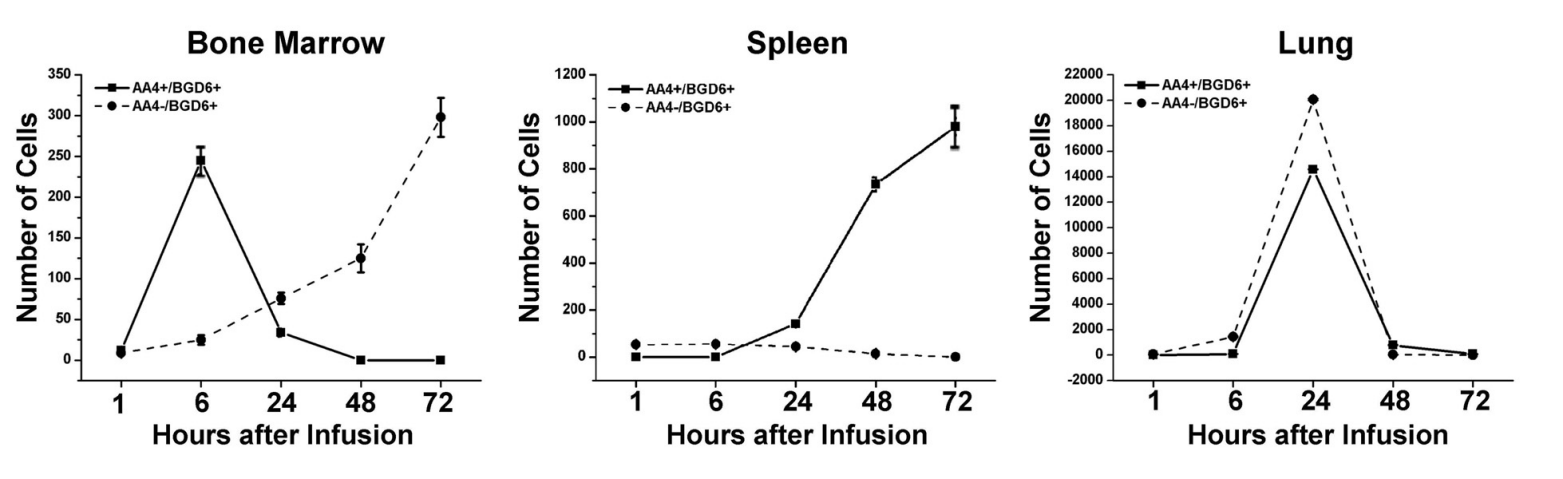
**MCcp home to the bone marrow **. MCcp (AA4-/BGD6+) and MCp (AA4+/BGD6+) were immunomagnetically isolated from the bone marrow and labelled with CellTracker Green CMFDA™ or CellTracker Red CMTPX™ respectively. The labelled cells were then infused into the tail vein of rats and at various times after infusion bone marrow (A), spleen (B) and lungs (C) were collected. The entire tissue was dissociated and the presence of labelled cells analyzed by FACS. The data is expressed as the number of labelled mast cells/10^5 ^cells. The average of 3 independent experiments ± SD is shown.

When the peritoneal cavity is depleted of mast cells, the data presented above suggests that it is the MCp (AA4+/BGD6+) that is recruited to the peritoneal cavity and that the MCcp (AA4-/BGD6+) remains and proliferates in the bone marrow. Therefore, it was of interest to investigate the homing patterns of the MCcp (AA4-/BGD6+) as well as the MCp (AA4+/BGD6+). The two populations of mast cells were immunomagnetically isolated and labeled with CellTracker™ Red or CellTracker™ Green. The labeled cells were then infused into the tail vein of rats. At various time intervals, cells from the bone marrow, spleen and lungs were examined by FACS for the presence of the infused cells. At 72 hours after infusion, the MCcp (AA4-/BGD6+) was concentrated in the bone marrow, while the MCp (AA4+/BGD6+) was concentrated in the spleen. At early times after infusion the MCp pass through the bone marrow and at 24 hours after infusion both MCcp and MCp are seen to transit through the lungs (Fig.8). These results confirm that the MCcp (AA4-/BGD6+) is addressed to the bone marrow.

**Figure 9:**
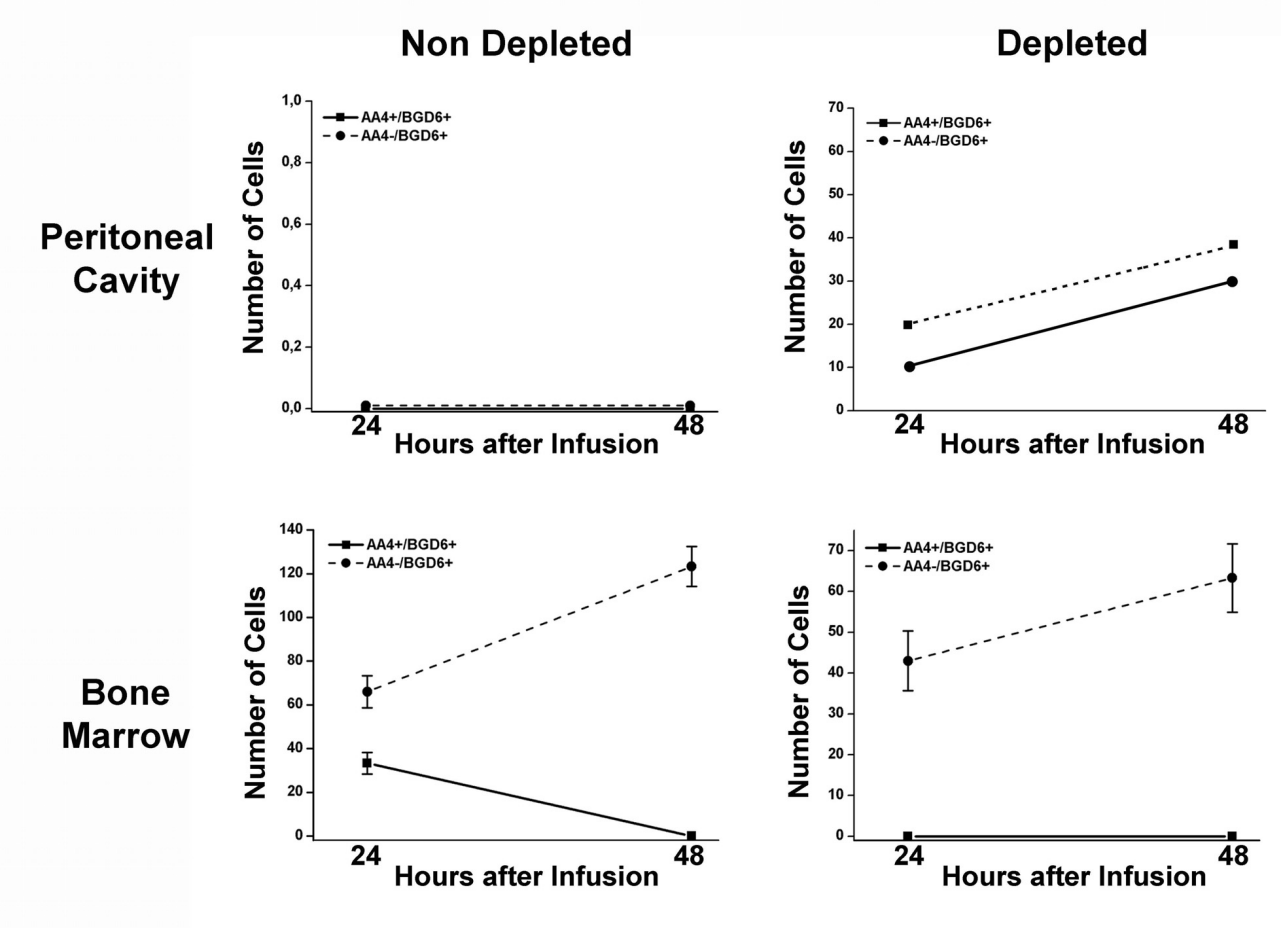
**When the peritoneal cavity is depleted of mast cells, both AA4-/BGD6+ and AA4+/BGD6+ cells go to the peritoneal cavity, but only the MCcp (AA4-/BGD6+) home to the bone marrow **. MCcp (AA4-/BGD6+) and MCp (AA4+/BGD6+) were immunomagnetically isolated from the bone marrow and labelled with CellTracker Green CMFDA™ or CellTracker Red CMTPX™ respectively. The cells were infused into non-depleted and depleted rats. At 24 and 48 hours after infusion, the peritoneal lavage and bone marrow were collected and examined by FACS for the presence of labelled mast cells. The data is expressed as the number of labelled mast cells/10^5 ^cells. The average of 3 independent experiments ± SD is shown.

The behavior of these two mast cell populations was then investigated during the initial phase of repopulation of the peritoneal cavity when mast cells are seen in the blood (Fig. 9). When the peritoneal cavity is not depleted, no infused cells are observed in the peritoneal lavage. As expected, under these conditions, the MCp (AA4+/BGD6+) only pass through the bone marrow, while the MCcp (AA4-/BGD6+) accumulates there. In contrast, when the peritoneal cavity is depleted of mast cells by distilled water both populations of mast cells are seen in the peritoneal lavage. At 48 hours after distilled water, while no infused MCp (AA4+/BGD6+) could be found in the bone marrow, there was a significant accumulation of MCcp in the bone marrow (Fig. 9). Therefore even under conditions of mast cell depletion, the infused MCcp still homes to the bone marrow, while the MCp goes preferentially to the peritoneal cavity.

## Discussion

This study demonstrates that bone marrow derived mast cells are involved in the repopulation of the peritoneal cavity following mast cell depletion. The MCcp (AA4-/BGD6+) is retained in the bone marrow and does not circulate, but proliferates and apparently gives rise to MCp that circulate and participate in the repopulation of the peritoneal cavity.

Mast cells arise in the bone marrow as a committed precursor that leaves the bone marrow as a MCp and completes its differentiation at peripheral sites [4-6]. The rat MCcp identified in this study is virtually identical to the MCcp present in Balb/c mouse bone marrow [15]. The MCcp from both species ranges from 3 μm to 4 μm, has a large, centrally located nucleus, scant cytoplasm, and by electron microscopy contain no cytoplasmic granules. The MCcp from both rats and mice is BGD6+, AA44-, FcεRI-, CD34+, CD13+, c-*kit*+, and does not bind IgE. A mast cell (Lin-, c-kit+, Sca-1-, Ly6c-, FcεRIα-, β7+,T1/ST2+) similar to the MCcp characterized in the present study has also been identified in bone marrow from adult C57BL/6 mice. The bone marrow derived mast cell from C57BL/6 mice gives rise only to mast cells when cultured and can reconstitute c-kit mutant mast cell deficient mice [29].

Little is known about the factors that may be responsible for release of mast cells from the bone marrow and their recruitment to peripheral sites. In the present study, two days after mast cell lysis by osmotic shock, degranulation by Compound 48/80 or anti-IgE the number of mast cells in the bone marrow was the same, indicating that mediator release by all three methods is equally effective in reducing mast cell numbers in the bone marrow. While mast cell lysis is thought to release only pre-formed mediators, activation of mast cells by Compound 48/80 or activation through FcεRI releases not only the pre-formed mediators stored in secretory granules, but stimulates the immediate production of newly formed lipid mediators as well as synthesis and release of cytokines and growth factors [30,31]. Weller et al., [32] using cultured mouse bone marrow mast cells reported that leukotriene B_4_, present in supernatants from activated mast cells, was the factor responsible for migration of immature mast cells and that other activation products were ineffective as chemotactic factors. However, since distilled water causes an immediate lysis of mast cells, it is not clear which, if any, of the newly-formed mediators are released. It is more likely that the recruitment of mast cell progenitors to the peritoneal cavity is mediated by the pre-formed mediators present in the secretory granules. A possible candidate for this recruitment is TGFβ1. Among the cell types involved in an innate immune response are mast cells [33]. When activated by Compound 48/80, rat mast cells have been shown to liberate TGFβ1. The TGFβ1 is stored in a latent form in secretory granules and upon mast cell activation is converted to the active form by chymase 1, which is also stored in the secretory granules [34]. Evidence suggests that TGFβ1 may be one of the most potent attractants for mast cells. TGFβ1 has been shown to cause directed migration of cultured mouse mast cells at femtomolar concentrations [35]. In the same study, other known mast cell chemoattractants including laminin, stem cell factor, and IL-3 were all found to be considerably less effective in inducing mast cell migration. In addition to the direct effect of mediators on mast cells, mast cell recruitment from the bone marrow could also occur through the indirect effect of mast cell mediators on other cell types [3,36-39], such as neutrophils, eosinophils, macrophages and fibroblasts. Activation of these other cell types could stimulate recruitment of MCp from the bone marrow.

Mast cell repopulation of the peritoneal cavity occurs not only through recruitment of MCp, but also by proliferation of newly recruited MCp. Under normal, non-pathological conditions, the mast cell mitotic index is considered to be very low [40], but in the present study, 48 hours after i.p. injection of distilled water, the percentage of mast cells in mitosis was seen to increase four fold. The use of mast cell specific antibodies coupled with immunostaining for phospho- histone H3 was essential for the identification of dividing MCp that do not yet contain metachromatic granules.

A previous investigation from our laboratory has shown that while mast cells freshly isolated from rat bone marrow express the integrin subunits α4, α5, α6, β1 and β7 on their surface, the more mature, adherent mast cells expressed significantly more α4 integrin [41]. This change in expression of α4 integrin may be related to the release of MCp from the bone marrow to populate peripheral sites. Specificity of integrin expression is known to play a pivotal role in mast cell homing. Gurish et al. [12], have shown that α4β7 integrin, but not αEβ7 integrin is required for tissue specific homing of mast cells to the small intestine. In β7 deficient C57BL mice, although mast cells were present in the lung, spleen, bone marrow and large intestine, mast cells were almost completely absent from the small intestine. Furthermore, in Balb/c mice that had been sub-lethally irradiated, administration of anti-α4β7 integrin, anti-α4 or anti-β7 antibodies blocked the mast cell repopulation of the small intestine after bone marrow reconstitution.

## Conclusions

The present study shows that in response to mast cell depletion of the peritoneal cavity, it is the MCp that is released from the bone marrow into the circulation in order to repopulate peripheral sites. Moreover, the MCcp is retained in the bone marrow and it is the MCcp population that expands to provide additional mast cells to repopulate these peripheral sites. This increased understanding of mast cell recruitment and repopulation provides additional therapeutic targets for control of the mast cell population in pathological conditions.

## Methods

### Animals

Young (150 g) male and female Wistar rats were used. Animals were housed and experiments were approved and conducted according to FMRP-USP, Ribeirão Preto, Brazil guidelines (protocol: 019/2005). For all experiments, animals were sacrificed by CO_2 _inhalation.

### Cells

Bone marrow was removed from the femurs of the rats by flushing the marrow cavity with Dulbecco's phosphate buffered saline (PBS) containing heparin (1000 units/mL (Produtos Roche Químicos e Farmacêuticos S. A., Rio de Janeiro, R.J., Brazil) 1% BSA (Sigma-Aldrich, St. Louis, MO) and DNase (1000 U/mL, Type I, Sigma-Aldrich). The cells were dissociated by aspiration with a Pasteur pipette, passed through a 25 μm nylon filter and then rinsed twice by centrifugation (27 × *g*) in PBS.

Blood was collected by cardiac puncture using a heparinized syringe.

Peritoneal cells were obtained by injecting rats i.p. with 15 mL PBS. The peritoneal wash was collected following laparotomy using a Pasteur pipette. The cells were rinsed twice by centrifugation (27 × *g*) in PBS and placed on Cell-Tak™ (BD Biosciences, San Jose, CA) coated coverslips.

### Antibodies

mAb AA4 (recognizes mast cell specific α-galactosyl derivatives of the ganglioside GD1b), mAb BGD6 (a mast cell lineage marker [15,20], and mAb BC4 (binds to the α subunit of FcεRI) were made in mice against the cell surface of RBL-2H3 cells, a rat mast cell line [16]. An affinity purified mouse mAb anti- rat IgE was purchased from Invitrogen, BioSource, Camarillo, CA, and an affinity purified mouse mAb anti- CD34 (ICO115) was purchased from Santa Cruz Biotechnologies, Santa Cruz, CA. A goat polyclonal antibody against CD13 was purchased from Santa Cruz Biotechnologies. The rabbit polyclonal antibodies against c-kit (Santa Cruz Biotechnologies) and against phospho-histone H3 (anti-phospho-histone H3 (Ser 10) (Millipore, Billerica, MA) were obtained commercially. Donkey anti-mouse IgG, anti-rabbit IgG or anti-goat IgG F(ab')_2 _fragments conjugated to FITC or Texas Red^® ^(Jackson ImmunoResearch, West Grove, PA) were used as secondary antibodies. For electron microscopy, donkey anti-mouse IgG conjugated to horseradish peroxidase (Jackson ImmunoResearch) was used as the secondary antibody. For some experiments antibodies were directly conjugated to FITC using the FluoReporter^® ^FITC Protein Labeling Kit or to Texas Red^® ^using the FluoReporter^® ^Texas Red^®^-X Protein Labeling Kit (Invitrogen, Molecular Probes, Camarillo, CA). Controls consisted of omitting the primary antibody or substituting the primary antibody with IgG from the same species. All controls were negative.

### Depletion and Degranulation of Peritoneal Mast Cells

Animals had their peritoneal cavities depleted of mast cells by i.p. injection of 15 mL of distilled water. Peritoneal mast cells were degranulated *in vivo *by i.p. injection with 4 mL of compound 48/80 (10-50 μg/mL; Sigma-Aldrich) in 0.02 M Tyrode-Tris buffer, pH 7.2 [42] or by i.p. injection of 1 mL containing 1, 2.5, 5, 10 or 15 μg/mL mAb anti- rat IgE (Invitrogen, BioSource) in PBS. Normal rat IgG (10 μg/mL, i.p.; Jackson ImmunoResearch Laboratories, West Grove, PA) served as a control.

At 6 and 10 days after distilled water injection, the peritoneal lavage was collected in sterile PBS, washed and aliquots placed on Cell-Tak™ (BD Biosciences) coated coverslips. The mast cells were sensitized for 1 hr with IgE anti-TNP (kindly provided by Reuben Siraganian, NIDCR, NIH, Bethesda, MD; 1:5000 in HBSS), rinsed in PBS and stimulated with 50 ng HSA-DNP_48 _(Sigma-Aldrich) for 30 minutes. In some experiments the mast cells were stimulated with Compound 48/80 (1 μg/mL; Sigma-Aldrich ) or mAb BC4 (0.5 μg/mL). After stimulation the cells were fixed in 4% formaldehyde in PBS, stained with toluidine blue (4% formaldehyde, 0.1% toluidine blue and 1% acetic acid, pH 2.8 for 10 min), and mounted on glass slides with Permount (Thermo Fisher Scientific Inc., Waltham, MA). Slides were examined and images acquired using an Olympus BX-50 microscope (Olympus America Inc., Melville, NY) coupled to a Nikon DXM-1200 digital camera (Nikon USA, Melville, NY)

### Collection and preparation of mesentery fragments

The duodenum was exposed and fragments of the mesentery were carefully detached from the intestine using surgical scissors. For light microscopy, the mesentery fragments were placed in a Petri dish, washed 3 times with PBS, spread over Styrofoam sheet in a Petri dish and held in place with plastic pins. The fragments were then fixed in 2% formaldehyde in PBS for 6-7 seconds in a conventional microwave oven [43]. After fixation, the mesentery fragments were washed in distilled water, and spread on glass slides. Samples to be stained with toluidine blue were dried on a heating plate at 45°C. After drying, adipose tissue was dissected away.

### Cell Separation

mAb BGD6, mAb AA4 or normal mouse IgG (Jackson ImmunoResearch) were conjugated to tosylactivated Dynabeads (Invitrogen, Dynal, Camarillo, CA) as previously described [44]. The cells were isolated according to the method of Jamur et al. [15]. Immunomagnetic isolation with mAb AA4 was used to obtain pure populations of granulated mast cells (AA4+/BGD6+) while sequential isolation with mAb AA4 followed by mAb BGD6 was used to isolate a pure population of MCcp (AA4-/BGD6+).

### Cell Counts

Mast cells were isolated with magnetic beads conjugated with mAb AA4 or mAb BGD6. After isolation, the number of cells either free or attached to the magnetic beads was determined by counting the cells in a hemocytometer. One thousand cells/antibody were counted in each of 25 experiments. In order to confirm the results obtained by immunomagnetic isolation, the percentage of mast cells that was present in the peritoneal lavage or bone marrow was determined in a separate set of experiments by counting the positive and negative cells after immunostaining with mAb BGD6, mAb AA4, or anti-IgE. A minimum of 1000 cells/antibody was counted in each of 25 experiments. The percentage of metachromatic mast cells in the peritoneal cavity was also determined by staining the peritoneal lavage with toluidine blue. All methods gave identical results.

The percentage of cells positive with mAb BGD6 or attached to the mAb BGD6 conjugated magnetic beads was taken as the total mast cell population. The percentage of MCcp (AA4-/BGD6+) mast cells was determined by counting the number of cells from the starting cell suspension that attached to mAb BGD6 beads after clearing twice with mAb AA4 beads. Five hundred cells attached to mAb BGD6 beads were counted for each of 41 experiments. The results were confirmed by immunostaining suspensions of bone marrow cells with both mAb AA4 and mAb BGD6. For cell separation experiments the number of cells in the peritoneal lavage or total bone marrow suspension was counted and the number of mast cells present was calculated based on the percentage of mast cells in the total bone marrow population as determined above (2.6 ± 0.5%). The number of MCcp (AA4-/BGD6+) cells present in the total bone marrow suspension was also calculated using the percentage (0.02 ± 0.007%) obtained above. The percentage of mast cells present in the bone marrow was determined by counting the positive and negative cells after immunostaining with either mAb AA4 or anti-IgE as well as by counting the number of cells that were either free or attached to mAb AA4 conjugated magnetic beads.

### Light Microscopy

Cells were rinsed in PBS and placed on coverslips coated with Cell-Tak™. In some experiments, cells were fixed and stained with toluidine blue for 10 min or mesentery fragments were fixed and stained with toluidine blue for 15 min. After staining the samples were washed quickly in distilled water, dehydrated and mounted in Permount (Thermo Fisher Scientific Inc.). For fluorescence microscopy, cells were placed on Cell-Tak™ coated coverslips, rinsed 3 times in PBS and fixed in 2% formaldehyde in PBS, or fixed in 2% formaldehyde for 10 minutes and permeabilized with Triton X-100 (phospho-histone H3). Mesentery fragments, fixed as previously described in a microwave oven, were permeabilized for 5 min in acetone at -20°C. The cells or mesentery fragments were rinsed in PBS, PBS containing 0.1 M glycine and incubated with mAb AA4 (5 μg/mL) or mAb BGD6 (20 μg/mL) directly conjugated to FITC or to Texas Red^®^, or anti c-kit (15 μg/mL), anti-CD13 (20 μg/ml), mAbCD34 (15 μg/ml), anti-IgE (5 μg/mL), or anti-phospho-histone H3 (5 μg/mL) for one hour at room temperature. Following incubation, the cells were rinsed thoroughly in PBS and if needed, the cells were further incubated with secondary antibody. All cells were then rinsed and the coverslips mounted with Fluoromount-G (EM Sciences) and observed by bright field, fluorescence and DIC microscopy using an Olympus BX-50 microscope (Olympus America Inc.) coupled to a Nikon DXM-1200 digital camera (Nikon USA,) or by scanning confocal microscopy (Leica Microsystems, Heidelberg, Germany).

### Electron Microscopy

For routine electron microscopy, cells were fixed in 2% glutaraldehyde (Ladd Research Industries, Burlington, VT) - 2% paraformaldehyde (Ladd) in 0.1 M cacodylate buffer, pH 7.4, containing 0.05% CaCl_2_, for 40 min at room temperature. Because all of the antigens detected are sensitive to glutaraldehyde, for immuno-electron microscopy the samples were fixed by microwave. Cells were suspended in 5 mL of fixative containing 2% formaldehyde, 0.05% glutaraldehyde (Ladd Research Industries; Burlington, VT), 0.025% CaCl_2 _in 0.1 M cacodylate buffer, pH 7.4 and irradiated for 4 seconds at 100% power [43]. Immediately after irradiation, 10 mL of PBS was added to the suspension and the cells were centrifuged for 2 minutes (27 × *g*). The cells were then rinsed twice in PBS, PBS containing 0.1 M glycine, and then in PBS + 1% BSA. Cells were incubated with anti- rat IgE (5 μg/mL) diluted in PBS + 1% BSA for 1 hour at room temperature. After incubation the cells were rinsed sequentially in PBS + 1% BSA, PBS, PBS + 1% BSA and then incubated for 1 hour with donkey anti-mouse IgG conjugated to horseradish peroxidase (25 μg/mL; Jackson ImmunoResearch) diluted in PBS + 1% BSA. The cells were then rinsed in PBS + 1% BSA, PBS and 0.1 M cacodylate buffer (pH 7.4) and immersed in diaminobenzidine incubation medium (12.5 mL cacodylate buffer, 12.5 mg diaminobenzidine (Polysciences, Inc, Warrington, PA) and 250 μl 1% H_2_O_2_) for 30 minutes at room temperature. The cells were then rinsed 10 times in cacodylate buffer. Some samples were packed by centrifugation in 1.5% agar before processing for electron microscopy. Cells were post-fixed in 2% OsO_4 _(EM Sciences) for 1 hour at room temperature, rinsed in distilled water, dehydrated through a graded series of ethanols, rinsed in acetone and embedded in Embed 812 (EM Sciences). Thin sections were cut with a diamond knife and stained for 10 minutes each in Reynolds' lead citrate [45] and uranyl acetate. Cells incubated without primary antibody or with normal mouse IgG (5 μg/mL; Jackson ImmunoResearch) in place of the primary antibody served as controls. None of the controls were immunolabeled.

### Proliferation assay

Forty-eight hours after distilled water injection, the peritoneal lavage was double stained with anti-phospho-histone H3 (5 μg/mL) to detect proliferating cells and with mAb AA4 to identify mast cells. A total of 2,500 mast cells from 10 different fields from 3 separate experiments were counted.

### Homing Assay

MCcp (AA4-/BGD6+) were labelled with CellTracker Green CMFDA™ (Invitrogen, Molecular Probes) and more mature mast cells (AA4+/BGD6+) were labelled with CellTracker Red CMTPX™ (Invitrogen, Molecular Probes). Cells were incubated for 30 min at 37°C in Iscove's medium (Invitrogen, GIBCO) with the fluorescent cell tracers (25 μg/mL). After incubation, the cells were rinsed and suspended in Iscove's medium supplemented with 10% fetal calf serum and incubated for an additional 30 min at 37°C. Cell viability, as determined by trypan blue dye exclusion, was >95%. The labelled cells (2.5 × 10^5 ^in 500 μL PBS) were injected into rats via the tail vein. The controls animals received only vehicle. In some experiments, the peritoneal cavity was depleted of mast cells with distilled water prior to injection of labelled cells. Tissues were collected from the non-depleted animals at 1, 6, 24, 48, and 72 hours after cell infusion and from the animals that had had their peritoneal cavity depleted of mast cells at 24 and 48 hours after infusion. The entire bone marrow, spleen, and lung were dissociated mechanically, the cell suspension homogenized using Pasteur pipettes and rinsed by centrifugation (27 × *g*) in PBS. Peritoneal cells were obtained by injecting rats i.p. with 15 mL PBS and collecting the lavage. The cell suspensions were washed twice by centrifugation (27 × *g*) in PBS and analyzed by flow cytometry using a BD FACS Calibur (BD Biosciences; Bedford, MA) with the CELLQuest program.

## Authors' contributions

MCJ participated in the conception, design and coordination of this study and helped draft the manuscript. ANM carried out the studies on mast cell repopulation of the peritoneal cavity. LFCM participated in the studies on the recruitment of mast cells from the bone marrow. DAS, Jr. designed and carried out the studies on mast cell homing. MRCC participated in the studies to phenotypically characterize the mast cell populations. MVDP carried out studies on mast cell proliferation and repopulation of the mesentery. ACGG carried out studies on mast cell recruitment to the mesentery and peritoneal cavity. DCS participated in studies on mast cell proliferation. CO participated in the conception, design and coordination of this study and helped draft the manuscript. All authors read and approved the final manuscript.
